# An ex vivo model of medical device-mediated bacterial skin translocation

**DOI:** 10.1038/s41598-021-84826-1

**Published:** 2021-03-11

**Authors:** Hao Wang, Anant Agrawal, Yi Wang, David W. Crawford, Zachary D. Siler, Marnie L. Peterson, Ricky T. Woofter, Mohamed Labib, Hainsworth Y. Shin, Andrew P. Baumann, K. Scott Phillips

**Affiliations:** 1 Center for Devices and Radiological Health, Office of Science and Engineering Laboratories, Division of Biology, Chemistry, and Materials Science, United States Food and Drug Administration, Silver Spring, USA; 2 Center for Devices and Radiological Health, Office of Science and Engineering Laboratories, Division of Biomedical Physics, United States Food and Drug Administration, Silver Spring, USA; 3 Perfectus Biomed Group (Formerly Extherid Biosciences, LLC), Jackson, WY USA; 4 Lubrizol Advanced Materials, Inc., Cleveland, USA; 5 Novaflux Inc, Princeton, USA; 6 Center for Devices and Radiological Health, Office of Science and Engineering Laboratories, Division of Applied Mechanics, United States Food and Drug Administration, Silver Spring, USA

**Keywords:** Antimicrobials, Assay systems, Biomimetics, Industrial microbiology, Bacterial infection, Fungal infection, Experimental models of disease, Preclinical research, Medical research, Pathogenesis, Materials science, Biomaterials, Diseases, Infectious diseases, Disease prevention, Public health

## Abstract

The skin is a barrier and part of the immune system that protects us from harmful bacteria. Because indwelling medical devices break this barrier, they greatly increase the risk of infection by microbial pathogens. To study how these infections can be prevented through improved clinical practices and medical device technology, it is important to have preclinical models that replicate the early stages of microbial contamination, ingress, and colonization leading up to infection. At present, there are no preclinical ex vivo models specifically developed to simulate conditions for indwelling medical devices. Translocation of pathogens from outside the body across broken skin to normally sterile internal compartments is a rate-limiting step in infectious pathogenesis. In this work, we report a sensitive and reproducible ex vivo porcine skin–catheter model to test how long antimicrobial interventions can delay translocation. Skin preparation was first optimized to minimize tissue damage. The presence of skin dramatically decreased bacterial migration time across the polyurethane catheter interface from > 96 h to 12 h. Using visual colony detection, fluorescence, a luminescent *in vitro* imaging system, and confocal microscopy, the model was used to quantify time-dependent differences in translocation for eluting and non-eluting antimicrobial catheters. The results show the importance of including tissue in preclinical biofilm models and help to explain current gaps between in vitro testing and clinical outcomes for antimicrobial devices.

## Introduction

**Figure 1:**
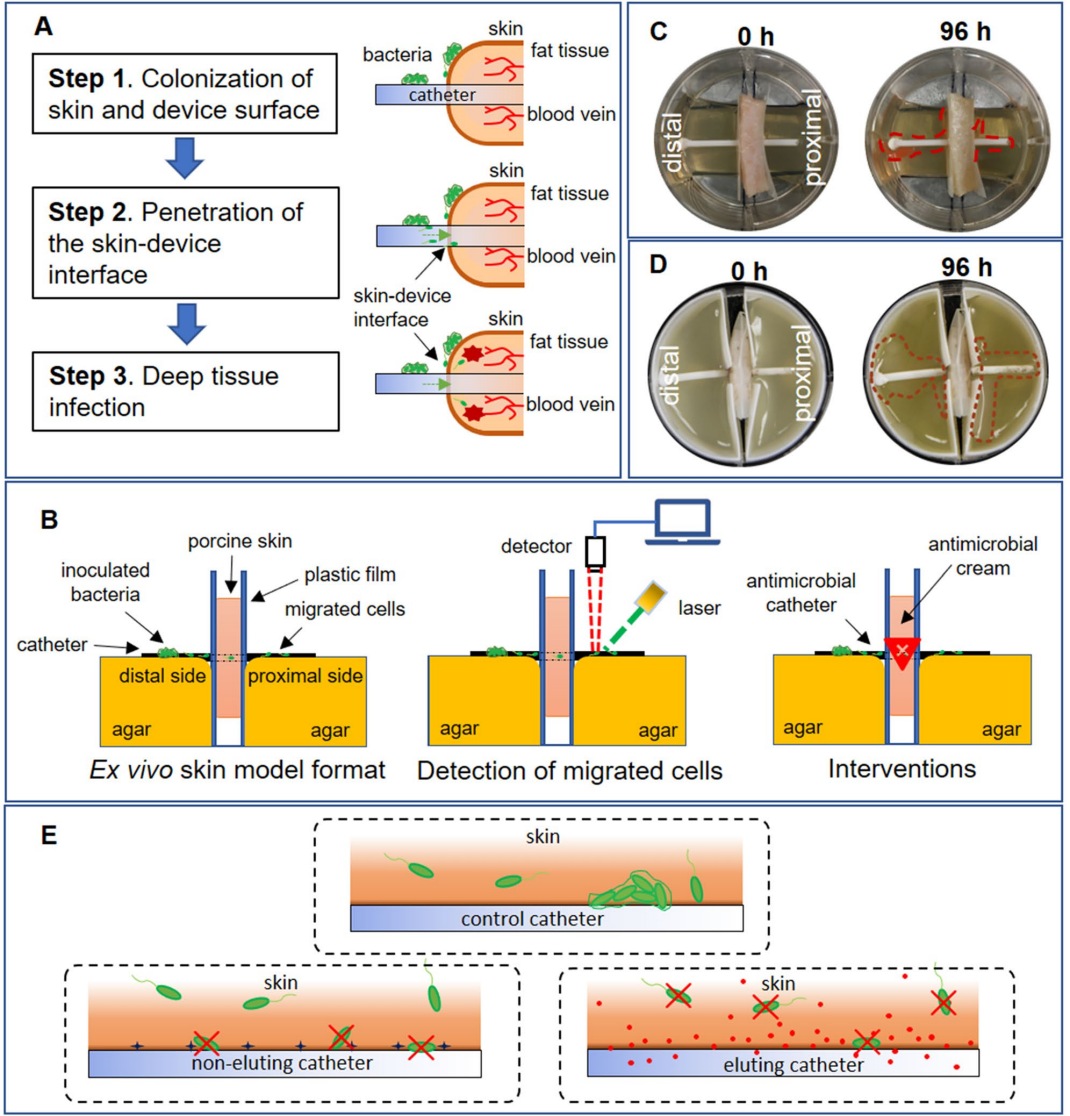
Ex vivo model to simulate pathogenesis of indwelling device-associated infection. (**A**) Proposed route by which bacterial cells penetrate the skin-device interface from superficial to deep tissue. (**B**) Schematic of ex vivo model. Bacterial cells inoculated on distal side of skin-device interface migrate to proximal side. Migration was monitored by fluorescence microscopy, confocal microscopy, and IVIS bioluminescence. Different interventions are evaluated to determine the comparative time of microbial translocation. (**C**) Photographic images of ex vivo model. Left: Initial inoculation of *E. coli* RP437/pRSH103. Right: *E. coli* RP437/pRSH103 biofilm on distal and proximal sides of agar 96 h post-inoculation (**D**) Photographic images from of ex vivo model using 3D-printed molds. Left: Initial inoculation of *E. coli* Xen14. Right: *E. coli* Xen14 96 h post-inoculation (areas of growth roughly outlined in dotted red line for clarity). (**E**) Schematic showing three scenarios studied in this work: bacterial interactions with tissue-control catheter interface, tissue-non-eluting catheter interface, and tissue-eluting catheter interface.

The skin is a primary barrier of the immune system, and it protects us from harmful microbes. Medical device injection and insertion—and especially continuous indwelling presence—compromises the skin and increases the risk of infection by microbial pathogens. The presence of central venous catheters (CVCs) is associated with infection rates as high as 5% per day^1^, while use of urinary catheters is associated with an approximate 1.5% infection rate per day^2^. Indwelling osseointegrated implants present an infection risk of 34–66% depending on different iterations of design and rehabilitation protocols^3–5^. Numerous pathogenic strains associated with infections have been isolated from contaminated indwelling devices. Among Gram-positive strains, *Staphylococcus aureus* (*S. aureus*) and *Streptococcus* spp. are often found in association with orthopedic devices or CVCs^4,6^, while Gram-negative *Escherichia coli* (*E. coli*) is the most common bacterium associated with urinary catheter-associated infection^7^. The association between location of an indwelling device and types of organisms found suggests that the skin microbiome plays a significant role^8^. Cells that colonize the soft tissue surrounding indwelling devices may passively or actively migrate on device surfaces or adjacent tissue, guided by chemotactic gradients^9^, colonizing the compromised superficial skin^4^ and, in more severe cases, infecting normally sterile internal organs and tissues (Fig. 1A).

To better study how these infections can be prevented through improved clinical practices and medical device technology, it is important to have preclinical models that replicate the early stages of microbial contamination, ingress, and colonization leading up to infection. Preclinical in vitro testing (sometimes called “bench testing”) is used throughout the medical device development pathway^10^. It serves as a rapid, least-burdensome approach to screening libraries of compounds or design features and is often evaluated in regulatory review as an antecedent to more expensive animal and human studies, helping minimize the ethical and financial impacts. Most testing used to study bacterial interactions with medical devices is based on existing pharmacologic assays such as minimal inhibitory concentration (MIC)^11^ or zone of inhibition testing^12^, or is based on biofilm reactors with various formats and endpoints (e.g., CDC biofilm reactor^13^, Calgary microplate assay^14^, or Certika assay^15^). Some of these assays are part of consensus standards for study of antimicrobial effects on bacteria and biofilms. For indwelling medical devices, no such standard method exists. One promising example is the in vitro bladder model developed by Darouiche et al.^16^ This model is designed to quantify biofilm formation on the internal lumen of catheters. It recapitulates the physiology and geometry of a urinary catheter, including use of artificial urine, to study antimicrobial effectiveness at the internal lumen of catheters. Since the model does not address the external lumen of catheters as a primary route of infection, it is necessary to further test devices in animal^17^ and human studies^18^ to confirm effectiveness. Another model developed by Sabbuba et al. measured migration of bacteria over urinary catheters^19^. Unexpectedly, the authors found that silver incorporated in hydrogel coatings on the catheters did not inhibit migration. Swarmer cells were able to migrate effectively, and could also transport other species. The model did not take into account the effect of the skin-catheter interface on migration, and did not quantitatively measure migration rates.

Unfortunately, antimicrobial performance measured using in vitro models often does not correlate well with clinical ability to prevent infections^20^. One key limitation of most preclinical models used to study indwelling devices is the conspicuous absence of tissue as an in vivo factor. Tissue in contact with medical device surfaces provides an alternate pathway for migration (indwelling devices) or colonization (implants)^21^. Compromised components may present a biologic signal to stimulate bacterial growth and virulence factors such as enterotoxins, leukocidins, and leukotoxins^22^. Tissue can adsorb antimicrobials through sequestration or binding, as well as absorb them into the matrix, resulting in suboptimal inhibitory concentrations and reduced bactericidal efficiency^23^. Bacteria also have adhesins for tissue surfaces and can produce necrotizing factors that damage tissue, encouraging biofilm formation^24^.

The use of tissue for preclinical testing could better simulate the in vivo environment at medical device interfaces. Ex vivo models—those that use animal tissue in an external environment—have greater control of variables, lower variability, and higher throughput than animal models^25^. Ex vivo microbiological tissue models have been developed to reduce animal testing, such as a human lung tissue model for pneumonia^26^ and a genital mucosal model for HIV infection^27^. Biofilm on ex vivo tissue is more challenging to eradicate^28–31^ and may better reflect pathogenic processes found in vivo than plastic plates or flow cells. For indwelling medical devices, the use of an ex vivo model also has unique potential to simulate the initial stages of bacterial skin translocation, which is not addressed by existing in vitro models.

In this work, we developed and characterized an ex vivo porcine tissue model for medical device-mediated bacterial translocation. We first optimized the preparation of skin by cleaning with an entangled microfibrillated cellulose network (NP paste) developed by NovaFlux to mechanically remove existing flora and contamination while minimizing tissue damage. Histology and MTT were used to evaluate the effects on tissue. Next, we developed a six-well plate format to increase testing throughput (Fig. 1B). 3D-printed inserts were designed to compartmentalize tissue held between two agar slabs. Tissue was biopsy punched and intravascular catheter tubing with a sealed opening was inserted and inoculated. The migration of bacteria was monitored using multiple endpoint methods. Results from visual inspection were compared with those from fluorescent microscopy, confocal imaging, and real-time luminescence tracking in an in vitro imaging system (IVIS). We validated the consistency of our results by repeating testing at two separate institutions. To demonstrate the utility of the method for evaluating antimicrobial medical device performance, two forms of polyurethane catheters with the same antimicrobial agent were tested—one non-eluting and one eluting. The antimicrobial stays on the non-eluting catheter, while on the eluting catheter the antimicrobial is slowly released into the device-tissue interface by diffusion. Since there is a need to improve prediction of *in* vivo performance and tissue presents a much greater challenge for non-eluting devices in vivo than in plastic models, we compared the results for these two catheter types tested with this method to a conventional in vitro method, the Certika assay. We also performed histology and viability assays on tissues exposed to the antimicrobial catheters to show that the model can be used for integrated, single-study benefit-risk analysis in early-stage technology development.

## Results

### Microorganisms cultured from ex vivo porcine skin after preparation

**Figure 2:**
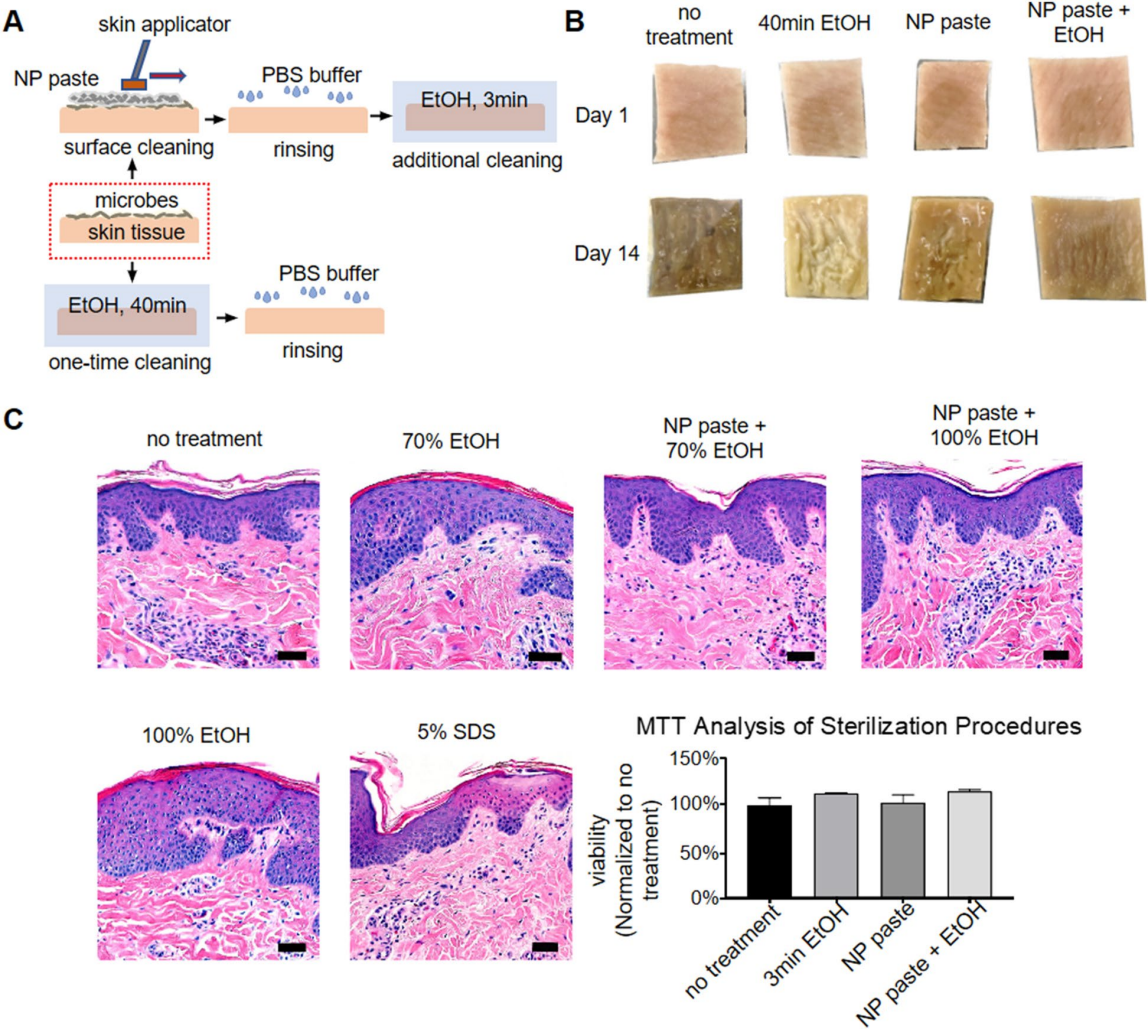
Porcine skin preparation process. (**A**) NP paste was applied to the skin surface and spread by moving an applicator gently back and forth for 5 min followed by sequential rinsing in PBS, 3 min incubation in 70% or 100% EtOH, and a final rinse in PBS. (**B**) Photographic images of skin at Day one and Day 14 after preparation. (**C**) Skin irritation analysis. Images: H&E stained micrographs (378×) for skin preparation compared with 40 min exposure to known irritants 5% SDS and 100% ethanol (Scale bar = 100 μm). Graph: MTT analysis of steps in skin preparation compared with no-treatment control. MTT analysis is graphed as mean ± SEM, and there were no statistically significant differences between groups (GraphPad Prism).

The ex vivo skin was prepared in several ways to determine the most effective sterilization protocol that did not result in visible skin irritation as observed by histology. These data were compared to untreated control skin that was rinsed with PBS buffer and superficially scratched with an inoculation loop and plated. Microbial growth appeared on tryptic soy agar (TSA) after day one, and on TSA plus chloramphenicol plates by day two (Table 1). This no-treatment control skin decayed rapidly (Fig. 2B). Samples collected from skin pre-cleaned with NP paste showed no growth on TSA plus chloramphenicol plates until approximately eight days, at which point they began to show visible signs of decay. Samples collected from skin soaked in 70% ethanol (EtOH) for 40 min and skin pre-cleaned with NP paste followed by a short 100% EtOH soak had no microbial growth of microorganisms on TSA plus chloramphenicol plates for up to 13 days (Table 1). Samples from skin pre-cleaned with NP paste followed by a 3 min soak in 70% EtOH yielded no organisms on TSA without chloramphenicol after five days of testing, at which point the skin sample was discarded. Analysis of samples collected from NP paste pre-treated skin with next generation sequencing showed *Bacillus amyloliquefaciens* was the major species that survived on the surface of skin after preparation. The results of histologic analysis (Fig. 2C) showed flattening of the stratum corneum (likely due to pressure applied during application) in both the sample treated with NP paste application and the sample treated with NP paste followed by a 3 min soak in 100% ethanol. None of the samples showed histologic evidence of tissue damage compared to the PBS control. The MTT data validate this finding, since percent viability was similar in treated samples compared to no treated PBS control.

**Table 1:** Day of culturable microbial growth on TSA agar.

Preparation	Plain TSA (day)	Chloramphenicol TSA (day)
No treatment	1 ± 0.0	2 ± 0.0
70% EtOH	2 ± 0.0	12 ± 1.0
NP paste	3 ± 0.0	8 ± 1.0
NP paste w/70% EtOH	> 5	NA
NP paste w/100% EtOH	4.5 ± 0.5	12.5 ± 0.5

### In vitro Certika biofilm assay of antimicrobial catheters

There are currently limited options for testing both eluting and non-eluting antimicrobial medical devices using the same method. Non-eluting materials typically don’t show measurable zones of inhibition. Consensus methods such as ISO 22,196/JIS Z2801 often used to test the biocidal activity of antimicrobial materials require a flat film for analysis^32^. Elution kinetics depend on geometry and thus three dimensional medical devices are not accurately compared using flat coupons. One in vitro assay capable of testing complex three dimensional geometries is the Certika assay, which is used by industry for early-stage research and development^33^. The assay monitors the kinetics of proliferation of daughter cells from the surface of the material after a short (1 h) challenge. A delay in the proliferation of daughter cells is indicative of a decrease in the number of bound cells, thus increased antimicrobial activity, and is quantified based upon the time delay for OD_578_ to reach 0.2. Antimicrobial samples are compared to a control material that does not contain any antimicrobial activity. Assuming one division every 30 min, a 6 h or 8 h delay in onset (OD_578_ = 0.2) when compared to the control equates to about a 3 log or 4 log reduction in bound bacteria respectively on the antimicrobial sample. The eluting and non-eluting catheter samples presented here were both analyzed using the Certika assay where the control material had an onset of 2.6–4.7 h, and both antimicrobial materials had a delay in onset > 48 h, demonstrating a > 4 log reduction.

### Visual observation of ***E. coli*** migration on skin-catheter model

RP437/pRSH103 *E. coli* colonies visible to the naked eye were established on the distal side of all skin-catheter models by 24 h, although there was repression of growth on the skin in contact with the eluting catheter. At around 48 h, colonies appeared on the proximal side of skin in the model with the unmodified catheter (See Supplemental Information, Figure S1). Neither the antibiotic control nor catheters inserted in the model without porcine skin (unmodified, eluting, and non-eluting) had visible colonies on the proximal side at the longest timepoint measured (96 h).

To confirm the assay was reproducible in skin from different sources and among different laboratories, experiments were conducted both at FDA and at Extherid Biosciences (Figures S2). FDA fabricated 3D-printed sample holders to improve the reproducibility between labs. Using these 3D-printed molds, Extherid obtained similar results with XEN14 *E. coli*: growth on the proximal side of the samples with unmodified catheter at around 48 h, growth on the proximal side of the samples with non-eluting catheter at 24 h, and no proximal growth with the eluting catheter (see Figure S2).

### Migration of red fluorescence protein (RFP)–producing ***E. coli*** on skin-catheter models

**Figure 3:**
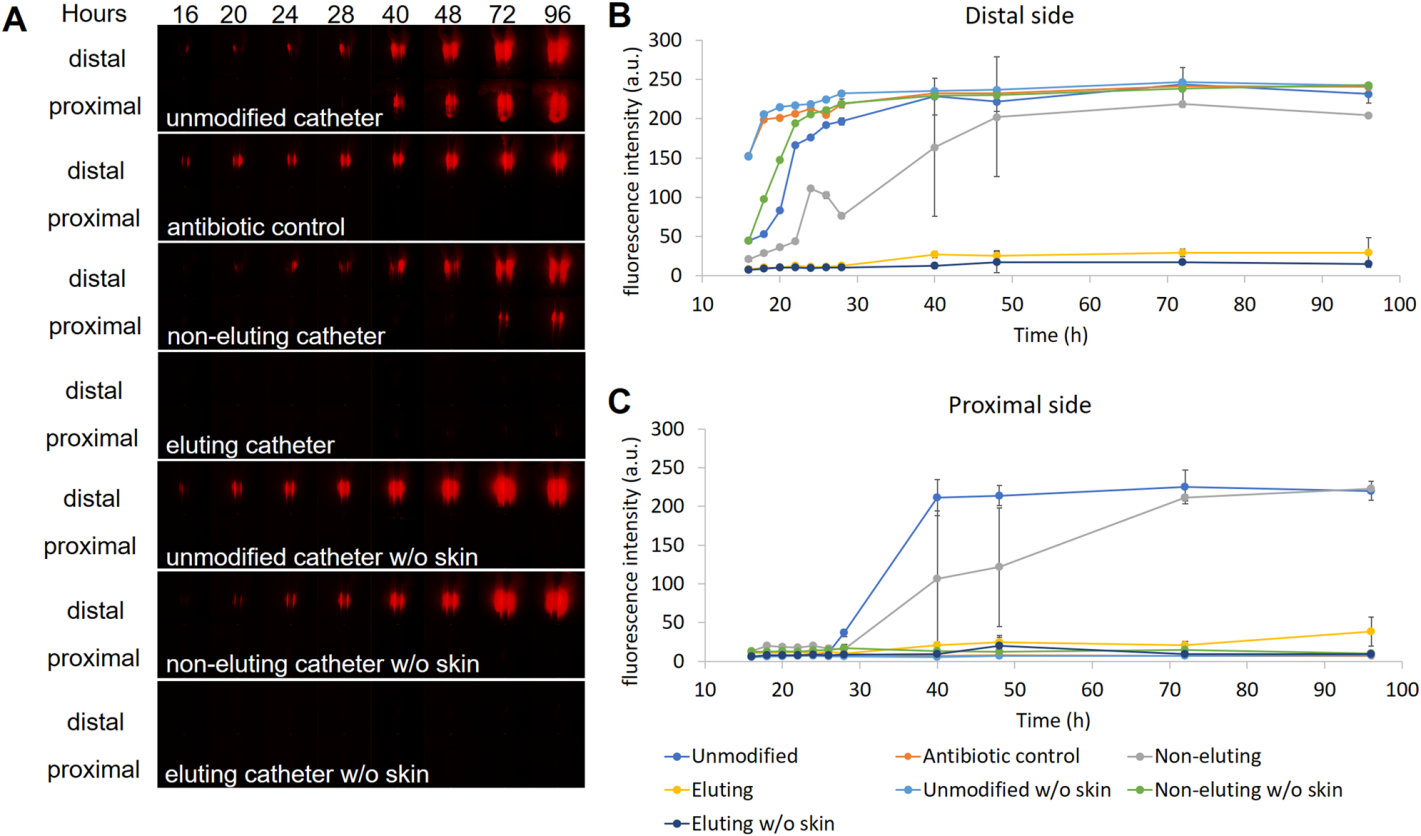
Migration of RP437/pRSH103 *E. coli* in ex vivo models at 16, 20, 24, 28, 40, 48, 72, and 96 h of incubation. (**A**) Fluorescence images of *E. coli* on both proximal and distal sides. (**B**) Time-dependence of integrated *E. coli* fluorescence intensity on the distal side. (**C**) Time-dependence of integrated *E. coli* fluorescence intensity on the proximal side.

*E. coli* RP437/pRSH103 colonies grew on the distal end of skin with unmodified catheters during the first 24 h after inoculation (Fig. 3A). Bacteria penetrated the interface between skin and catheter and fluorescence was detected on the proximal side at 28 h after inoculation. The samples with skin pretreated with antimicrobial ointment (antibiotic control) on both sides (but not on the agar or catheter) were also tested to represent a treatment with a known result, which was also an effective intervention to prevent indwelling catheter associated infection^34^. Growth of colonies on the distal side was not affected by the antibiotic ointment, and no fluorescence was observed through the 96 h endpoint on the proximal side. The non-eluting catheter had detectable fluorescence on the proximal side after 48 h. Eluting catheters showed no visible colony growth on the distal side through the endpoint, and there was also no fluorescence on the proximal side through the endpoint. Catheters inserted in the model without porcine skin (unmodified, eluting, and non-eluting) had normal colony growth on the distal side but did not have fluorescence on the proximal side through the endpoint.

The integrated fluorescence intensity was quantified over time (Fig. 3B,C). On the distal side, fluorescence from most of the samples was tightly clustered in the first 24 h, with a rapid increase from the initial value up to about 200–250 relative units (RU). On the proximal side, the time-dependent effects of antimicrobial interventions were clearly distinguished. The unmodified catheter showed the earliest increase in fluorescence (26 h) with the steepest slope. The maximum fluorescence value reached by 40 h (200–250 RU) was similar to that seen on the distal side. The non-eluting catheter showed a delay in the starting time (28 h) and a smaller slope of increase. By 72 h, fluorescence on the proximal side had reached a level similar to the unmodified catheter. By contrast, the proximal side of the antibiotic control and the proximal side of all the models without skin showed no increase in fluorescence through the endpoint.

There was a small increase in fluorescence for the eluting catheter (40 RU) on both proximal and distal sides, although visible colonies were not observed.

### Migration of luminescent Xen14 *E. coli* on skin-catheter models monitored by IVIS

**Figure 4:**
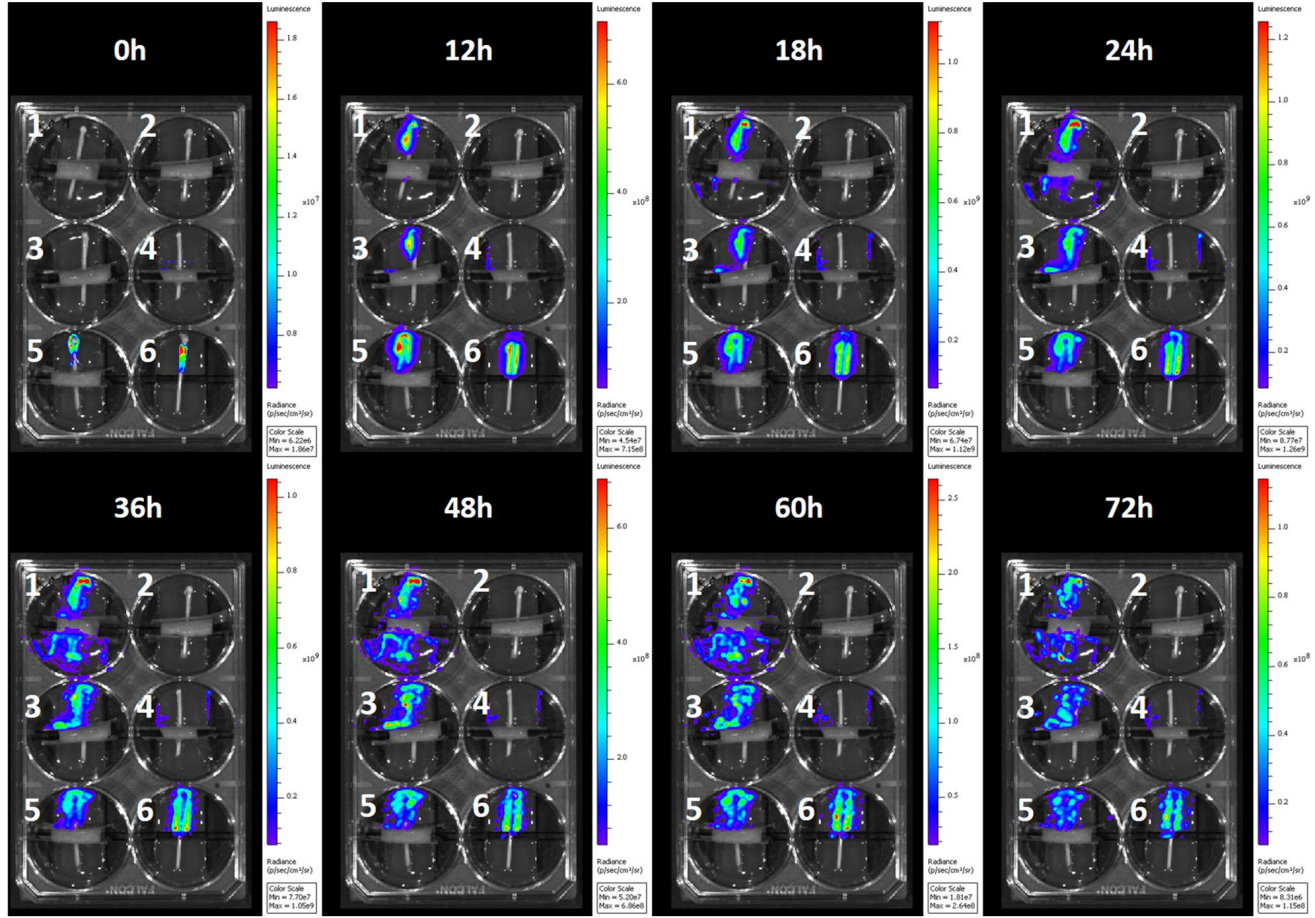
IVIS images of bioluminescent *E. coli* XEN14 on ex vivo model at time at 0, 12, 18, 24, 36, 48, 60, and 72 h post-inoculation. Real-time video (Video S1) showed migration path through the skin-catheter interface. Interventions: (1) Unmodified catheter with skin, (2) No inoculation with skin, (3) Non-eluting catheter with skin, (4) Eluting catheter with skin, (5) Antibiotic Control, (6) Unmodified catheter without skin.

The migration behavior of bacteria in ex vivo models was also observed in real-time with an IVIS luminescence imaging system (Fig. 4). Similar to the results seen with fluorescence detection at fixed timepoints, bacterial growth on the distal side was observed within 12 h on all catheters except the drug-eluting catheter. On the proximal side of the unmodified catheter, luminescence could be seen at 12 h after inoculation. For the sample antibiotic control, no luminescence was observed up to 72 h after inoculation. For the non-eluting catheter, an unexpected interaction between the antimicrobial compound and the luminescent protein appeared to quench luminescence. While bacteria could be observed by eye on the proximal side of the non-eluting catheter at longer time points, no luminescence was seen. Subculturing of these bacteria on fresh agar restored the bioluminescence. Careful analysis of the real-time data (**Video S1**) showed that the bacteria were not migrating along the side of the well plate or the top surface of the skin; therefore, the skin–catheter interface was the route by which the bacteria were reaching the proximal agar.

### Biofilm formation at skin-catheter interface imaged by confocal laser scanning microscopy

**Figure 5:**
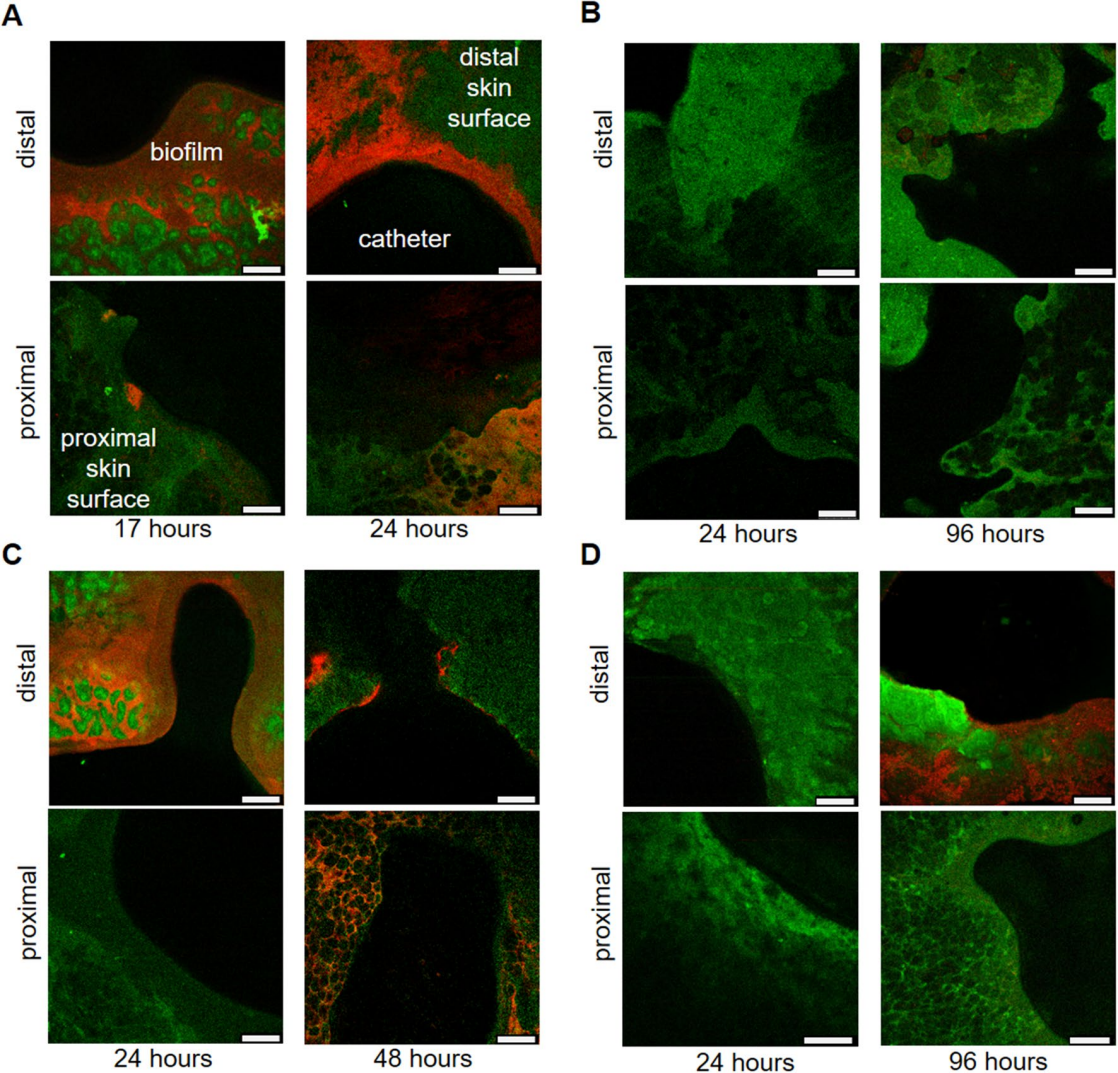
Fluorescence confocal laser scanning microscopy images of porcine skin’s distal and proximal surfaces that had been in contact with different catheters. Skin surface autofluorescence is green, RP437/pRSH103 *E. coli* is red, and the black region is insertion site of catheter. Images for each timepoint were taken of sections around the catheter, as seen by the curvature where the biopsy punch was made. Distal side is the epidermis and proximal side is mostly hypodermis and associated fat cells. (**A**) Skin around unmodified catheter, (**B**) Skin from antibiotic control, (**C**) Skin around non-eluting catheter, (**D**) Skin around eluting catheter. Scale bar = 50 µm.

To understand migration of bacteria at the skin-catheter interface and how this changed with different antimicrobial interventions, tissue samples were imaged with confocal laser scanning microscopy (CLSM) at several timepoints after inoculation (Fig. 5). The skin surrounding the unmodified catheter (Fig. 5A) showed significant *E. coli* (red) colonization of the epidermis in contact with the catheter (distal side) at 17 h, starting from the interface and spreading across the skin surface. The proximal side of the skin showed some colonization already beginning by that time. At 24 h, the distal side of the skin was heavily colonized and the proximal side (primarily hypodermis with visible fat) had increased colonization spreading outward from the interface. It appeared that the *E. coli* colonization was less robust on the fatty tissue than on the epidermis. This is consistent with the previous findings that fatty acid could prevent biofilm formation by inducing dispersion^35,36^. As expected from the results reported above, no colonization of the antibiotic control skin (Fig. 5B) was observed at 24 h. At 96 h, no visible *E. coli* colonies could be seen on the proximal or distal tissue (Fig. 5B). For the non-eluting catheter (Fig. 5C), there was slower migration, similar to what was observed by fluorescence. The distal side was colonized at 24 h, but not as heavily as with the unmodified control catheter. At 48 h, there was colonization on the edges of the distal side and on parts of the proximal tissue as well. For the eluting catheter (Fig. 5D), there was no colonization observed on either side at 24 h, while significant colonization spreading outward from the interface was seen on the distal side only at 96 h. Altogether, these data suggest the mechanism used by the bacteria to traverse the skin was by colonizing tissue in contact with the catheter, followed by migration to the other side of the skin through the interface.

### Histological analysis of antimicrobial interventions

To show that the model could be used simultaneously for both antimicrobial testing and topical biocompatibility assessment, we studied the tissue used in the ex vivo model using hematoxylin and eosin (H&E) staining. First, we assessed how different skin preparation methods affected the skin (Fig. 2C). Skin samples prepared with NP paste followed by 3 min submersion in EtOH contain intact apical and basolateral domains of epithelial cells, similar to the “no treatment” sample. Use of a commercially available skin applicator to scrub the skin likely caused some flattening of the stratum corneum due to mechanical force. The positive control (5% SDS, 40 min) had abnormal pink discoloration in the epithelium, bleaching of the epithelial apical domain, and pyknotic nuclei throughout the epidermis, all indicators of irritation.

**Figure 6:**
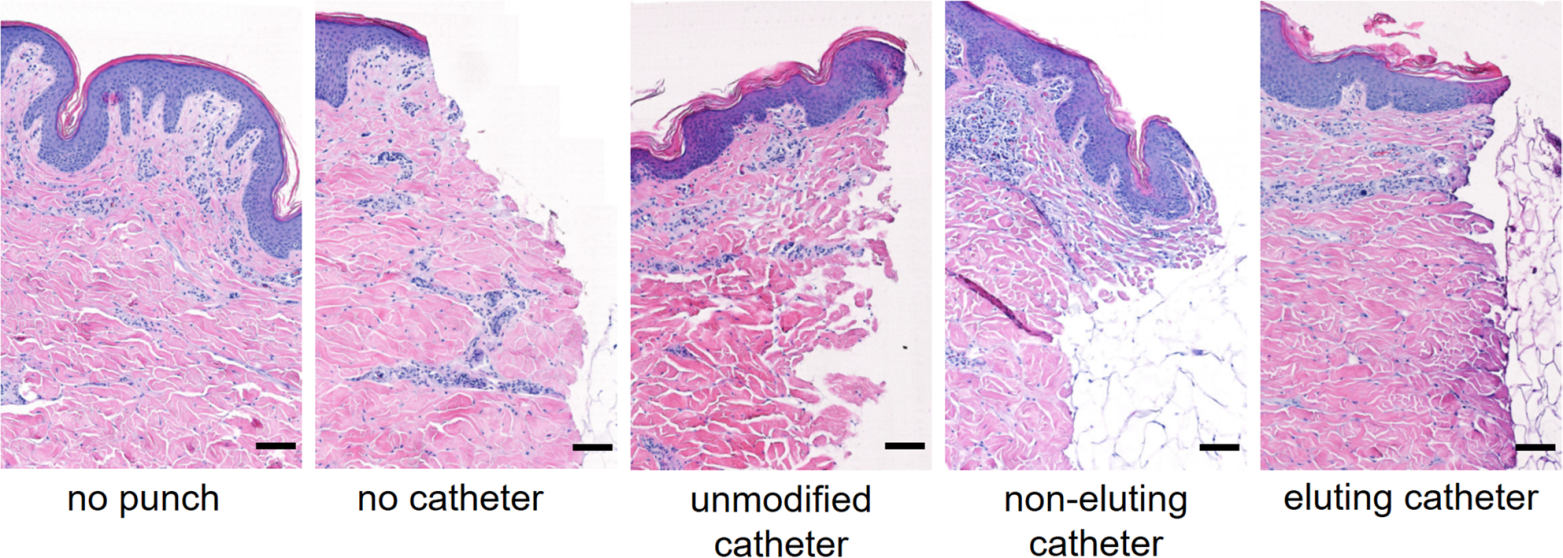
H&E stained tissue from 48 h ex vivo models visualized at 378×. In addition to conditions used above, a “no-punch” sample and “no-catheter” sample were used as reference controls. Images show a longitudinal cross-section with the skin surface on top and the skin-catheter interface on the right side of the image. Scale bar = 100 μm.

To study topical effects of indwelling catheters on the porcine skin, samples from porcine skin that had been in contact with the catheters for 2d under normal experimental conditions were stained with H&E (Fig. 6). There was no evidence of gross bacterial or fungal growth on any of the samples, confirming the results of culture testing that showed the skin preparation protocol was effective. The non-eluting and eluting antimicrobial catheters tested in this study did not induce any obvious histological abnormalities in the porcine skin. Slight discoloration of the eosin stain at the interface of the skin and the eluting catheter was noted, possibly indicating penetration of the antimicrobial in the tissue.

## Discussion

### The presence of tissue in the ex vivo model is a rate limiting factor in bacterial migration across catheter segments

Microbial biofilms present a significant challenge to many antimicrobial technologies due to hindered diffusion, binding to the matrix, the presence of persister cells, and more facile gene transfer^37–39^. Researchers have sought to incorporate the challenge of biofilms by testing new antimicrobials against them in place of planktonic cells. While this strategy can mimic biofilms on a hard surface or in lumens and pipes, it doesn’t capture the full challenge of preventing colonization in corporal medical devices. Recently, development of tissue-based biofilm models for studying wound treatments, and their use by an increasing number of contract testing organizations, has shown the potential of tissue-based preclinical models for testing biofilms. In this work, we leveraged experience with porcine skin models for injection^30^ and skin preparation^29^ to develop and characterize a model for indwelling medical devices.

Skin preparation is an essential first step in ex vivo models to maximize the useful test time, as well as minimize response variability due to contamination. While high concentrations of strong chemical disinfectants can effectively kill all contaminants on the skin, they may alter the skin structure and/or leave residuals that could interfere with assays. As an alternative to the use of strong disinfectant conditions, we used a micro-fibrillated cellulose cleaning paste (NP paste) developed by NovaFlux to generate frictional forces on the skin surfaces to assist removal of soil, including bacterial cells and biofilms (Fig. 2). This allowed for use of shorter and less aggressive EtOH treatment to achieve similar results. Using this approach, swabs from the skin cultured on TSA did not show growth for up to 4d, and swabs cultured on TSA plus chloramphenicol did not show growth for up to 13d. Histological slices stained with H&E also did not show microorganisms after 2d. The source of porcine skin can have a significant impact on contamination and usable time. We found that fresh skin obtained directly from the source had less contamination and longer usable time than processed skin. While the use of EtOH can potentially influence the skin barrier and native flora, it is commonly used before inserting catheters and thus helps mimic the clinical scenario while ensuring consistent results by killing any microbial contaminants that could interfere with the assay.

The format used for testing was also important to obtain reproducible results (Fig. 1). We focused on use of a 6-well plate to provide increased throughput over single-sample tests. Although we tested skin in the lateral plain with a vertical penetration, we found that this format made aspects of testing and detection more complex. By placing the skin vertically between two slabs of agar, the entire setup was easily accessible for manipulation and measurements and avoided potential gravity-driven migration through the skin-device interface. Initially, Parafilm was used to separate the skin from the agar. Using IVIS real-time monitoring of the migration process, we noted that bacteria sometimes took alternate migration pathways, resulting in variability of migration time. By replacing the Parafilm with a 3D-printed sample holder that held the skin precisely in place, we obtained more consistent intra- and inter-lab results.

Testing of catheters without skin showed that under the conditions of initial bioburden and humidity used in this work, bacteria did not traverse an approximately 3 mm gap between agar connected only by catheter tubing within the test period of 96 h (Fig. 3A). When skin was placed in the gap, bacteria rapidly traversed the same space in less than 26 h, as shown by a combination of the visible fluorescence data and confocal microscopy data for the unmodified catheter. This time is similar to the shortest incubation period known for catheter-associated urinary tract infection, which is 24–48 h after catheter placement^40^ (although the length of the device-tissue interface is much longer for an actual urinary catheter). The skin was easily colonized and appeared to promote bacterial migration and colonization, showing that the porcine tissue explant is a more challenging scenario when compared with abiotic models.

Quantitative fluorescence migration data showed that bacteria grew rapidly on the distal side of all samples except the eluting catheter (Fig. 3B). By the first time point measured after incubation, there was already a significant fluorescent signal on the distal side for the unmodified catheter and antibiotic control. By the 40 h timepoint, both the unmodified and antibiotic control catheter had a similar maximal level of fluorescence on the proximal side, with only a slight increase in fluorescence over the next 56 h. This maximum may have been reached due to bacteria reaching a stationary phase, either because the maximum density of cells that could colonize on the surface of skin-catheter model had been reached, or because some limitation in the agar nutrient supply had been reached.

On the proximal side (Fig. 3C), the unmodified catheter with skin had a rapid increase in fluorescence at 26 h, reaching a maximum by 40 h. Migration of luminescent/fluorescent bacteria was seen as early as 12–17 h in the more sensitive IVIS and CLSM formats. For the antibiotic control, no fluorescence increase was seen on the proximal side as late as 96 h. Fluorescence was observed along the distal end of the antibiotic control catheter but not on the skin surface at that time. The antibiotic control was the only sample for which no bacterial cells were seen at the assay endpoint (96 h) on the distal side by highly sensitive CLSM (Fig. 5). The results for the unmodified catheter and antibiotic control were consistent across all four formats (visible colonies, fluorescence, IVIS, and CLSM), although the point at which translocation was detected depended on the sensitivity of the methods. These visible colony results were also reproduced by a second laboratory (Extherid) which used the same format with similar reagents and bacterial strains.

### The performance of antimicrobial interventions in the ex vivo model is significantly affected by the presence of tissue

As an initial step in the process of infectious pathogenesis, migration across the device-tissue interface presents an important potential target for antimicrobial interventions. However, most existing assays for testing antimicrobial device performance are not designed to incorporate tissue or to measure skin translocation, but instead measure a reduction of microbes in solution, or the amount of microbes on a material at one or more timepoints. We first evaluated antimicrobial catheters with a common in vitro test used by industry, the Certika assay^15^. The assay measures repression in bacterial adhesion and subsequent propagation of daughter cells from the surface of materials. The unmodified catheter material showed an onset of growth around 2.6–4.7 h, whereas both the non-eluting and eluting antimicrobial materials had a significant delay in onset of > 48 h. The results from this assay suggested that both materials would have similar antimicrobial performance.

When the antimicrobial catheters were tested in the ex vivo model, the pattern and time frame for antimicrobial performance differed from the Certika assay. On the distal side, non-eluting antimicrobial catheters had an 8 h delay relative to unmodified catheters before fluorescence could be detected, although by 48 h they eventually reached maximal levels similar to the unmodified catheters (Fig. 3B). On the other hand, eluting catheters had minimal fluorescence on the proximal side throughout the test, reaching only 40RU at 96 h. No colonies were observed on the proximal side for the eluting catheters, so this signal was likely an artifact due to autofluorescence from the antimicrobial. These results show that the endpoint measurement time is a critical factor for in vitro antimicrobial assays. If we had stopped the assay at 8 h or even 24 h, the non-eluting catheter would have shown a reduction in bioburden compared to the unmodified control. Yet, by the second day, the non-eluting intervention no longer functioned to prevent migration and growth. This could be one reason why many short-term in vitro assays for biofilm do not correlate with clinical outcomes.

On the proximal side (Fig. 3C), the non-eluting catheter delayed the start of an increase in fluorescence until about 40 h and showed a more gradual increase in fluorescence than the unmodified catheter, reaching the maximum at 72 h. The CLSM images (Fig. 5) showed extensive colonization of the proximal skin by 48 h, consistent with the fluorescence results. The bacterial cells appeared to migrate to the proximal skin and begin colonization even while they were still increasing in number on the distal skin. This supports the role of necrotic skin as a nutrient rich and growth-stimulating source for the bacteria that allows it to overcome immune responses and antimicrobial interventions, which is also observed in chronic wound research^41–43^. By comparison, the eluting catheter showed only a small amount of fluorescence on the proximal side, which was likely due to autofluorescence from the antimicrobial elution. No visible colonies were observed on the distal side of the eluting catheter for up to 96 h. At 4d, fluorescence was seen in some confocal images of the distal skin for the eluting catheter. This may have been due to bacterial colonization or autofluorescence from the buildup of the antimicrobial in the tissue.

The difference in results between conventional and ex vivo assays is likely because of (1) the difference in the in vitro and ex vivo environment in these models and (2) the difference in the endpoint that was measured (bacteriostasis vs. translocation). The endpoints of conventional in vitro antimicrobial assays (e.g., zone/time of killing, MIC, or bacteriostasis) for biofilm are not correlated with clinical outcomes and do not simulate the in vivo environment or pathogenesis. While an extensive review of the literature is beyond the scope of this paper, multiple meta-analyses have concluded that many antimicrobial devices, many of which show promising in vitro results, have no effect on infection rates^21,44–46^. Although it is clear that adhesion, colonization, and biofilm formation play an important role in vivo, it is challenging to simulate this process for a number of reasons. The in vivo environment is difficult to recreate, and there are many chemical and biological forces acting at the device-tissue interface that are not well understood. On the other hand, skin translocation can be partially simulated with the use of ex vivo tissue or tissue culture. Since blocking translocation with an antimicrobial device can delay the onset of bacteria getting inside the body, it is more likely to delay infection. Once bacteria are inside a surgical site, they have many potential mechanisms to survive an antimicrobial device, such as growing in biofilm and colonizing necrotic tissue. The endpoint measured with the ex vivo model is the migration rate of bacterial cells through the skin-catheter interface, which is represented by the breakthrough time when visible colonies are detectable on the proximal side. Thus, the ability to measure an actual delay time for translocation with the ex vivo model is more likely to lead to a clinically relevant metric connected with outcomes for indwelling device-associated infection. The difference between 48 h protection and 8 h protection against migration could have a significant impact on clinical outcomes for some short-term indwelling-device applications. For example, in a two week catheter placement, the ability to prevent translocation for 4d could potentially reduce some infections that occur between day 10 and day 14.

### Unifying antimicrobial effectiveness with tissue damage testing in early stage development

Although the goal of antimicrobial strategies is to kill bacteria without harming tissue, there is usually a trade-off between effectiveness and cell toxicity. An example is silver nanoparticles, where it appears that cell toxicity occurs at levels below the effective antimicrobial concentration^21^. Often the formats used for antimicrobial effectiveness testing are different from those used for biocompatibility testing, which could impact the ability to compare data that is obtained with these preclinical methods. Some biocompatibility assays may be performed in culture media or in animal studies. They may require a non-standard sample size, geometry, or sample format that results in a different elution profile than would be expected in vivo. At the same time, it is often difficult to define a specific quantity to characterize eluting antimicrobial devices, since they have an elution profile that evolves over time. Achieving an identical elution profile for biocompatibility testing as that used for antimicrobial testing may be difficult due to specific requirements of many biocompatibility tests. A potential advantage of the ex vivo model is that it can be used for both effectiveness testing and also for evaluating tissue damage under identical conditions. For example, the method could be used to test a small library of catheters with increasing antimicrobial mass to determine the optimal trade-off between effectiveness and tissue damage.

To ensure that the method of preparing skin did not damage the tissue, both histologic evaluation and MTT assay were performed on fresh skin samples (Fig. 2). Although there was some minor flattening of the stratum corneum caused by mechanical scrubbing, this is likely a tradeoff required to remove entrenched microbial flora. No other abnormalities were seen for any of the individual preparation conditions or the process as a whole, while damage was seen for the control irritants (40 min 100% EtOH or SDS). To show the potential for evaluation of tissue damage by antimicrobials using the ex vivo model, we performed histologic evaluation of tissue samples that had been in contact with the unmodified, non-eluting, and eluting catheters (Fig. 6). No histologic abnormalities were found. Some unusual discoloration was seen for the skin in contact with the eluting catheter, which may be due to the interaction of eosin with the antimicrobial. Although the effects that can be observed on ex vivo porcine skin are limited, in future work it may be possible to obtain greater sensitivity in histologic analysis by using explanted human skin, which has been reported to stay alive in RPMI 1640 medium with normal human serum for at least 24 h^28^.

## Conclusions-limitations and future development

Further research with animal models will be helpful to establish correlation between results from the model and in vivo outcomes. Clinical data will also help validate the relevance and reliability of the model for potential regulatory use. The use of ex vivo tissue as a tissue source in this work could potentially be used up to two weeks with a fresh and clean tissue source. Longer-term testing will require the development of living tissue approaches that could be used in this format. Recent tissue culture constructs potentially make months-long testing possible. The use of fresh cadaver tissue or human skin donated after plastic surgery, bathed in culture medium, may also provide longer usability. The use of live skin also opens the door to study biological factors such as inflammatory mediators in the skin^47^. The health of the skin may be affected not only by antimicrobials and bacteria individually, but may be uniquely affected by the combination of both- which can be elaborated with this model. This could also enable the model to provide device-bacteria-tissue interface biocompatibility information that is not captured by current standard methods based on ISO 10993.

Additional work needs to be done to better understand how other variables affect results. Skin preparation removes potential immune factors such as antimicrobial peptides or beneficial organisms that are present on the skin surface. For the purpose of testing, the removal of these factors may help to increase uniformity across different tissue samples and create a “worst-case” scenario (in addition to the primary objective of removing organisms that would interfere with the testing process). As our understanding of the protective role of native flora increases, an approach to clean the skin and then restore a uniform density of protective flora to all skin coupons could be developed. Most indwelling devices also experience mechanical motion that could cause tissue compromise and increase the risk of translocation. A more complex model could incorporate the role of mechanical motion in testing.

As noted in the results, in the presence of antimicrobials, it is important to look for visible colonies and not rely solely on fluorescent or luminescent constructs. It is possible not only that antimicrobials can cause bleaching of photonic signals, resulting in a false negative measurement as observed here, but also that dead bacterial colonies can continue to retain photonic signal. An important advantage of the time-based assay used in this approach is that bacteria must be alive to migrate across the skin, thus eliminating the possibility of a false positive measurement due to the live/dead state of the bacteria itself. A false positive from the antimicrobial or its interaction with skin is a possibility, which should be mitigated through the use of appropriate controls and comparison of baseline values at matched timepoints.

Another advantage of the time-based format is that it is amenable to different types of antimicrobial strategies including chemical, physical, and combination strategies. The model can be used to study not only how well antimicrobials on a device prevent translocation, but also to better understand potential treatments of skin around the abutment, such as daily cleaning, probiotics, ultrasonic waves, electrochemical field, etc. This could be especially valuable for osseointegrating implants, which include orthopedics used for wounded warriors, products in clinical trials, as well as possibly adaptation for dental implants.

## Materials and methods

### Skin preparation and monitoring of residual microorganism growth on agar plates

Porcine skin used for experiments performed at FDA (approximately 64 cm^2^) were either sourced from a local market (Rockville, MD) or provided by Extherid Biosciences (Jackson, WY). Skin bought from local market was generally washed with clean water and stored at − 5 °C prior to use in experiments. Skin used for experiments performed at Extherid were sourced from a local farm and received 3–5 h after the pig was harvested for meat. Tissue was immediately processed by shaving and rinsing skin surface and removing excess subcutaneous fat and then stored in RPMI medium at 4 °C for 1–3d prior to use in experiments.

Immediately before experiments, skin samples were first washed by submerging in sterile 1× PBS (pH = 7.4); then, approximately 10 mL of NovaFlux Nano Clean paste (NP paste) (NovaFlux, Princeton, NJ) was applied on both sides of the skin followed by gentle scrubbing (~ 2 N) with an unactivated ChloraPrep applicator (BD, Franklin Lakes, NJ) (no chlorhexidine was squeezed out into the applicator tip) for 5 min. NovaFlux Nano Clean is a microstructured fluid with a 3D gel-like network of micro-fibrillated cellulose. It cleans the skin by mechanical interactions. Skin samples were again washed with 1× PBS and then soaked in either 100% ethanol or 70% ethanol for 3 min. Sterilized samples were stored in 1× PBS until the next step (Fig. 2A). Another selected preparation approach was soaking the skin samples in 70% ethanol for 40 min followed by rehydration in 1× PBS for 30 min (Fig. 2A).

To evaluate sterilization efficacy, skin samples that underwent the different preparation protocols were incubated at 30 °C, and the presence of superficial skin microbes was investigated every 24 h by scratching the surface of the skin with an inoculating loop and then inoculating TSA plates (nonselective TSA used for all samples, TSA with 10 µg/ml chloramphenicol for all samples except the NP paste/70% ethanol sample). TSA plates were then incubated at 37 °C for 2–3d, after which presence and level of microorganism growth was noted. The microorganism was subcultured in 3 ml TSB medium with 10 µg/ml chloramphenicol, and then the slant culture was sent to MicroGen Diagnostics (Lubbock, TX) for sequencing.

### Assembly of skin-catheter model

Thermoplastic polyurethane (TPU) intravascular catheters were cut into approximately 4 cm pieces and sterilized prior to use by soaking in 70% ethanol for 10 min. Skin was sterilized with NP paste and ethanol as described above. Sterilized skin was cut into pieces measuring approximately 1.5 cm × 1 cm × 0.3 cm and a hole was punched in the center with a 2 mm biopsy punch. One of three different TPU catheters (i.e., unmodified, or modified with an antimicrobial (either non-eluting or eluting); Lubrizol Advanced Materials, Cleveland, OH) was inserted into this hole with approximately 2 cm protruding on either side of the skin. The catheter diameter was about 1.33—1.66 mm (4–5 French Gauge), and the size of the punched hole in skin tissue was smaller than 2 mm due to tissue elasticity. The catheter came into contact with the tissue as it was inserted and no visible gaps were present. A piece of Parafilm approximately 2 cm × 1 cm with a 3 mm hole punched in the center was used to cover all but the skin-catheter interface. Next, the skin-catheter setup was inserted into a gap (approximately 3 mm) between two TSA blocks in a 6-well plate. The TSA used in this experiment was approximately 0.75% agar (relatively soft). The in vitro antibacterial effectiveness of catheters were accessed with the Certika proliferation assay (QualityLabs BT GmbH, Nuremberg, Germany)^15^.

This gapped-TSA setup was prepared in one of two ways: setups used in initial experiments were prepared by pouring soft TSA agar into an empty well and allowing it to harden. Prior to the experiment, an approximately 0.3 cm gap was cut in the center of the agar, the edges of the agar were trimmed away to prevent bacteria from migrating along the edge, and the trimmed agar was air-dried for about 2 h. The skin-catheter setup was placed in the center of the gap, the catheter ends were partially submerged in the TSA, and the Parafilm-covered skin was pressed against the TSA (Fig. 1B). The exposed plastic surface of the 6-well plate was covered with approximately 30 mg of triple antibiotic ointment to prevent contamination with bacteria.

To make the setup more convenient and consistent, FDA developed a 3D-printed mold that allowed agar to be poured directly into separated containers with a vertical gap to accommodate the catheter (Fig. 1D). Following placement of the skin-catheter setup in either apparatus, the proximal opening of the catheter was closed with silicone sealant to prevent bacteria from migrating through the interior of the catheter. For the antibiotic control, before inserting the catheter into the skin, the skin's whole surface was spread with ointment by a sterile cotton applicator and then punched in the central area by a 2 mm biopsy puncher.

### Simulation of contaminated skin and indwelling device in skin-catheter model

We modeled translocation of bacterial cells over the skin-catheter model with two *E. coli* strains: RP473/pRSH103 and XEN14. Organisms were streaked for isolation onto TSA plates supplemented with 30 µg/mL tetracycline or 10 µg/mL kanamycin, respectively. Before experiments, strains were subcultured in tryptic soy broth (TSB) supplemented with 30 µg/ml tetracycline or 10 µg/ml kanamycin at 37 °C to OD (600 nm) 0.9–1.0 (Extherid-specific bacterial preparation in Supplemental Information S2). Then 5µL of 3–4 × 10^8^ CFU inoculum was pipetted onto the end (distal side) of the catheter and adjunct agar. After the inoculum had dried, the skin-catheter model was incubated at 30 °C and bacterial colony growth was observed by eye and recorded by camera (Fig. 1C,D). To compare results with those yielded by an in vitro system, the antimicrobial effects of the TPU catheters used in the skin-catheter model were also evaluated by Certika assay in the absence of skin (Supplemental Information S3)^15^.

### Monitoring migration of fluorescent bacteria by long-working-distance microscopy

*E. coli* RP473/pRSH103 constitutively expresses red fluorescent protein, which makes it straightforward to track with fluorescent microscopy^48^. To track the movement of bacterial cells in the model, we fabricated a long-working-distance microscopy system that included a supercontinuum laser light source (SuperK EXTREME EXU-6, NKT Photonics, Denmark) operating at 553 nm excitation wavelength and a stereomicroscope (SZ-ST + SZ40 + SZ-CTV, Olympus, Waltham, MA) detecting fluorescence with a red bandpass filter (ET620/60 m, Chroma Technology, Bellows Falls, VT) and monochrome digital camera (PL-D734MU-NIR, Pixelink, Ottawa, Canada). The working distance was 5-20 cm, and exposure time for imaging was 800 ms. The skin-catheter models inoculated with *E. coli* RP473/pRSH103 were incubated at 30 °C. Fluorescence images of skin-catheter models were taken at room temperature at 16, 20, 24, 28, 40, 48, 72, and 96 h after inoculation and processed using channel tools and image stacks in ImageJ (National Institutes of Health, Bethesda, MD). No values were manipulated- only the display colors were changed. Fluorescence intensity of the biofilm around the catheter was quantified with MATLAB (R2018b, The MathWorks Inc., Natick, MA) by identifying the intensity value which was 99% of the maximum in the selected area around the catheter.

### Monitoring migration of bioluminescent bacteria by IVIS

*E. coli* migration in the skin-catheter model by means of a similar test with a bioluminescent *E. coli* strain and an IVIS (IVIS Spectrum, PerkinElmer, Waltham, MA). Skin-catheter models were inoculated with *E. coli* XEN14 and incubated at 35 °C in the IVIS chamber. Images were taken every hour with binning and 1 s exposure time in both luminescent and brightfield channels. The bioluminescence strength of the bacterial cells was monitored for 74 h.

### Confocal imaging of skin samples with bacterial migration at different timepoints

Imaging of *E. coli* RP473/pRSH103 biofilm on porcine skin surfaces at different time points was performed with confocal laser scanning microscopy (CLSM) (SP8, Leica Microsystems, Wetzlar, Germany). Porcine skin samples were removed from well plates and imaged directly after removal of catheters. CLSM images were collected in two channels: 395 nm excitation/405–430 nm emission for skin surface and 543 nm excitation/580–630 nm emission for *E. coli* biofilm.

### Histological analysis of antimicrobial interventions

Skin samples used for histological analysis were prepared as described above. To analyze the effects of various antimicrobial interventions (Fig. 2C), a 5 mm biopsy punch was used to create an explant from the skin sample immediately following treatment. The explant was placed in 500μL of neutral buffered formalin and stored at 4 °C until processing. Skin samples used to analyze the effects of in-dwelling catheters were washed in PBS, treated with NP paste for 5 min, soaked in 70% or 100% ethanol for 3 min, and washed with PBS. These samples were then prepared as if for a bacterial migration experiment, as described above. Skin samples were incubated at 30 °C for 2d, after which the catheter was removed and a 5 mm biopsy punch was used to remove an explant centered around the catheter site. Explants were placed in 500 μL of neutral buffered formalin and stored at 4 °C until processing. Formalin-fixed explants were embedded in paraffin and cut into approximately 5 μm slices with a microtome. Slices were stained with hematoxylin and eosin (H&E) and coverslipped. Stained slices were then visualized with a BX63 microscope (Olympus, Tokyo, Japan) at 378× magnification.

Samples were simultaneously collected for MTT analysis, a generally accepted method for estimating skin irritation^49^. Explants were made with a 5 mm biopsy punch and immediately placed in 100μL of MTT reagent, where they incubated at 37 °C for 3 h. Following MTT incubation, explants were destained in 100μL of 0.1 M HCl in 2-propanol overnight at 4 °C in individual wells of a standard 96-well microtiter plate. Explants were removed from destain solution, which was measured for absorbance at 570 nm. Data were normalized by dividing the 570 nm absorbance of each sample group by average 570 nm absorbance of the untreated control. Data were imported into Prism (Graphpad) and analyzed by one-way ANOVA with a Dunnett post-test for multiple comparisons.

## Supplementary Information

Supplementary Video 1.

Supplementary Information 1.
