# Supplementary material for: An ex vivo model of medical device-mediated bacterial skin translocation
Source: Sci Rep. 2021 Mar 11;11:5746. doi: 10.1038/s41598-021-84826-1 (PMC7952406; doi:10.1038/s41598-021-84826-1)
Supplement: Supplementary file 2 — Supplementary Information 1. [file 41598_2021_84826_MOESM2_ESM.docx]

**An *ex vivo* model of medical device-mediated bacterial skin translocation**

Hao Wang^1^, Anant Agrawal^2^, Yi Wang^1^, David W. Crawford^3^, Zachary D. Siler^3^, Marnie L. Peterson^3^, Ricky T. Woofter^4^, Mohamed Labib^5^, Hainsworth Y. Shin^1^, Andrew P. Baumann^6^ and K. Scott Phillips^1^

^1^Center for Devices and Radiological Health, Office of Science and Engineering Laboratories, Division of Biology, Chemistry, and Materials Science, United States Food and Drug Administration, Silver Spring, USA

^2^Center for Devices and Radiological Health, Office of Science and Engineering Laboratories, Division of Biomedical Physics, United States Food and Drug Administration, Silver Spring, USA

^3^Perfectus Biomed Group (formerly Extherid Biosciences, LLC), Jackson, WY, USA

^4^Lubrizol Advanced Materials, Inc., Cleveland, USA

^5^Novaflux Inc., Princeton, USA

^6^United States Food and Drug Administration, Center for Devices and Radiological Health, Office of Science and Engineering Laboratories, Division of Applied Mechanics

**Supplementary material**

**Supplemental information S1. Replication of catheter migration experiment (Extherid Biosciences)**

Skin and catheters were processed as described in **Materials and Methods,** with a minor variation in skin preparation: following NP paste treatment, skin section was soaked in 70% (rather than 100%) ethanol for 3 min. Skin-catheter model was assembled as described in **Materials and Methods** using 3D-printed molds provided by FDA and 0.75% TSA with 10µg/ml chloramphenicol. The experiment was performed as described in **Materials and Methods** with the following differences in bacterial preparation: the only strain used in the experiments performed at Extherid Biosciences was *E. coli* XEN14. *E. coli* was subcultured on nonselective TSA plates and grown in nonselective TSA medium prior to use in the experiment. The following conditions were analyzed in the experiment: 1) skin with XEN14 *E. coli* but no catheter to ensure that bacteria were not able to cross without a catheter, 2) skin and unmodified catheter but no added XEN14 *E. coli* to ensure that no growth originated from the skin, 3) skin that had been pretreated with approximately 30mg of triple antibiotic ointment with an unmodified catheter and XEN14 *E. coli* as a non-catheter-based antimicrobial intervention, 4) skin and unmodified catheter with XEN14 *E. coli*, 5) skin and noneluting catheter with XEN14 *E. coli*, and 6) skin and eluting catheter with XEN14 *E. coli.* The 6-well plate containing the samples was incubated at 30°C for 5d and observed every 24 h. Observation of bacterial migration at Extherid was performed by eye and documented by camera (Canon 60D with 17–85mm, f/4–5.6lens).

The results from the experiment performed at Extherid **(Figure S2)** are in general agreement with those obtained at FDA (**Figure S1**). In the well that did not contain a catheter but was infected with XEN14 *E. coli,* growth occurred on the distal side (site of infection) but not on the proximal side, indicating that growth on the proximal side was catheter dependent. No observable growth occurred in the well that was not infected with XEN14 *E. coli*, indicating that contamination by endogenous bacteria on the skin was not a concern. The well that contained an unmodified catheter, XEN14 *E. coli*, and skin treated with triple antibiotic ointment had growth on the distal but not the proximal side of the skin, indicating the sensitivity of this model to antimicrobial intervention.

The results of experiments performed with skin, XEN14 *E. coli*, and the three trial catheters were in general agreement with those at FDA. The well containing an unmodified control catheter had visible growth on the proximal side of the apparatus at 48 h, in line with FDA results. The well containing the eluting catheter had visible growth on the distal side of the catheter but none on the proximal side throughout the experiment. The only result that varied from those reported by FDA occurred in the well with the unmodified catheter, which had slight visible growth on the proximal side of the catheter by 24h (**Figure S2**). At FDA, proximal bacterial growth with the unmodified catheter took place considerably later (48–72 h, **Figure 3)**. This variation may have occurred due to differences in the physical condition of the skin in the skin-catheter assembly.

**Supplemental Information S2. Histologic analysis of sterilization protocol with 70% ethanol**

To isolate the effects of 70% ethanol vs. 100% ethanol treatment, a sample set that consisted of an untreated control, skin treated with 70% ethanol only, skin treated with NP paste only, and skin treated with both ethanol and NP paste was prepared in accordance with the **Materials and Methods**. The results of histologic analysis of the samples shown in **Figure S3** were consistent with the results with 100% ethanol shown in **Figure 2C**, which also showed the flattening of the stratum corneum in both the sample treated with NP paste application and the sample treated with NP paste followed by a 3min soak in 70% ethanol, and no histologic evidence of tissue damage compared to the PBS control.

**Supplemental Information S3. *In vitro* Certika proliferation assay**

The 200 µl of 5 x 10^6^ CFU/ml *E. coli* DSM1756 (ATCC8739) was incubated together with the catheter sample in the well at 37 °C for 1h. The sample was then rinsed to remove any non-specifically bound cells before it was transferred to the new well with minimum essential medium (1xPBS with 1% tryptic soy broth (TSB)), and allowed to incubate at 37 °C for 18h. Daughter cells propagated from the sample’s surface and then the sample was removed followed by adding 50µl TSB medium into the well. The daughter cells proliferated in the medium while monitoring optical density every 30 min for 48h. The antimicrobial activity was then assessed based on the growth curve.

**Supplemental figures**


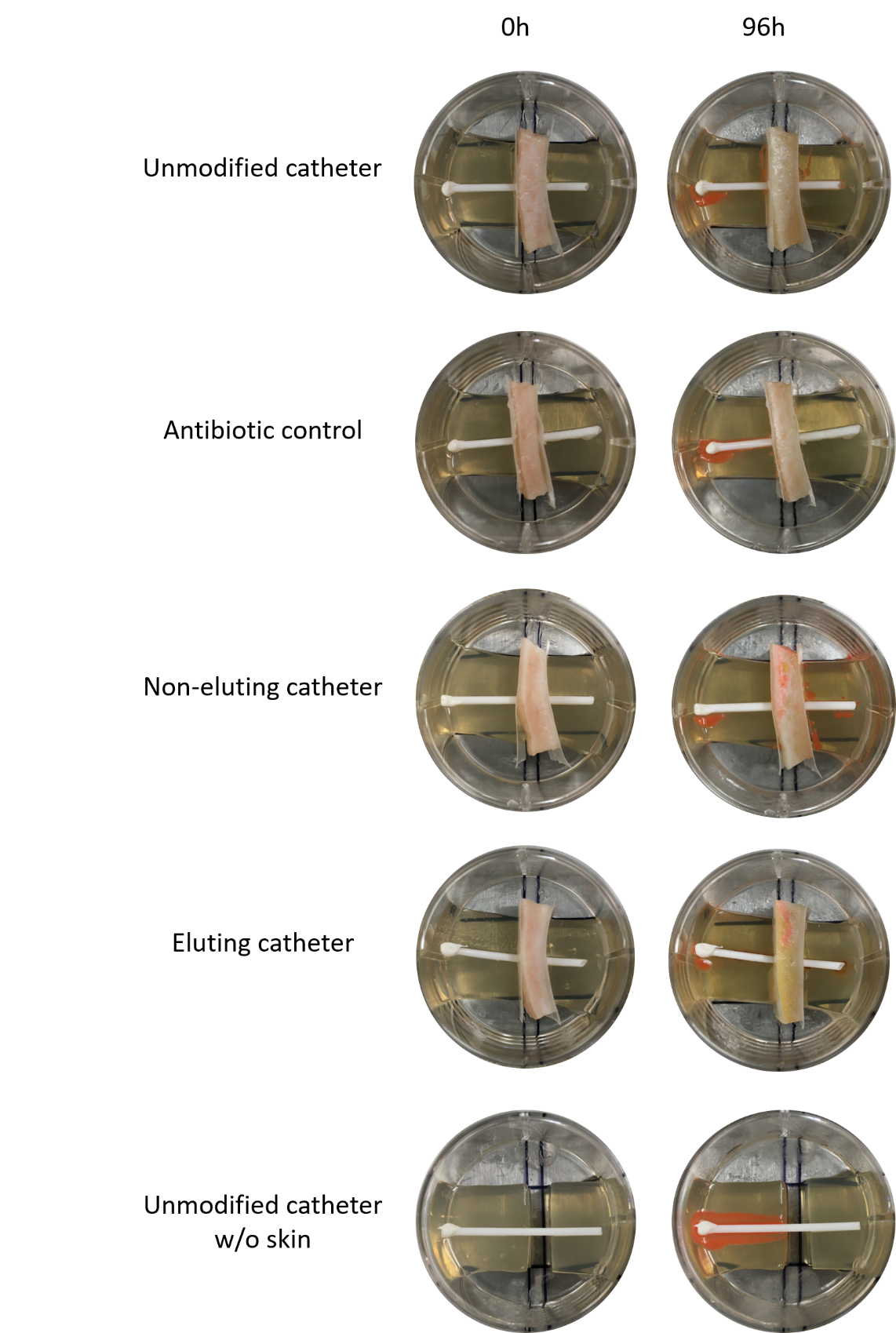


**Figure S1.** Photographic images of *E. coli* RP437/pRSH103 in *ex vivo* model with unmodified catheter, antibiotic control, non-eluting catheter, eluting catheter and unmodified catheter without skin at 0 and 96h.


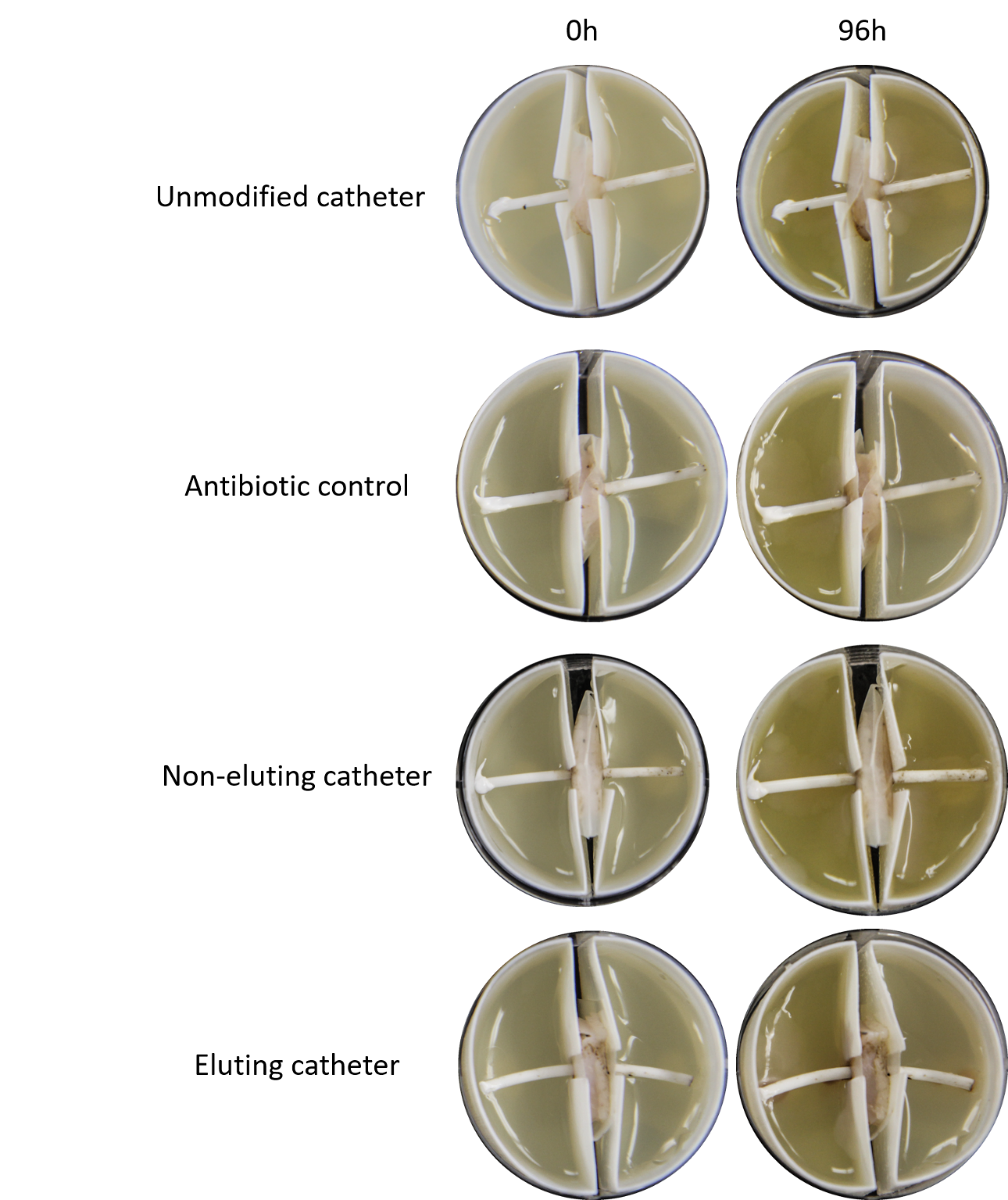


**Figure S2**. Photographic images of *E. coli* XEN14 in *ex vivo* model with 3D-printed molds and unmodified catheter, antibiotic control, non-eluting catheter or eluting catheter at 0 and 96h (Extherid). Approximate extent of bacterial growth on the proximal side is highlighted by red dashed line.


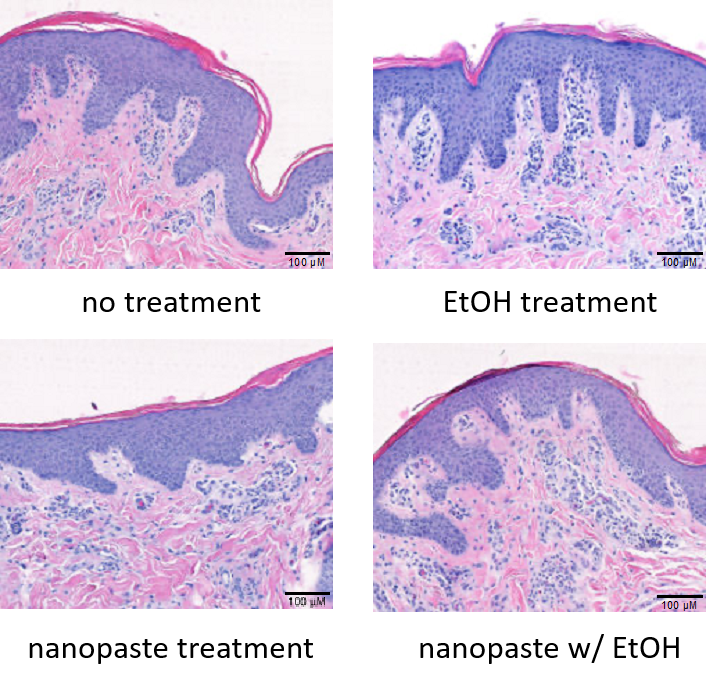


**Figure S3**. Histologic examination of skin samples to isolate the effects of 5min treatment with NP paste treatment and 3min submersion in 70% ethanol.

**Video S1**. Movie of *E. coli* XEN14 migration in *ex vivo* models with different interventions during 74h incubation in IVIS.
